# Modelling sovereign credit ratings and assessing the impartiality: A case study of China

**DOI:** 10.1371/journal.pone.0289321

**Published:** 2023-09-08

**Authors:** Min Su

**Affiliations:** College of Economics and Management, Taiyuan University of Technology, Taiyuan, China; American University in Dubai, UNITED ARAB EMIRATES

## Abstract

The post-COVID-19 era presents a looming threat of global debt, elevating concerns regarding sovereign credit ratings worldwide. This study develops a new index system, divides the rating variables into long- and short-term factors, performs rating fitting and prediction, and investigates the fairness of China and relevant countries. Our findings reveal that sovereign credit ratings have a deterrent effect on the global financial market due to the ceiling effect and quasi-public goods characteristics. A high and stable credit rating demands long-term enhancements in economic fundamentals, budget balances, external surpluses, and overall solvency. Concurrently, effective short-term debt management strategies, including reduction, repayment, and swaps, are essential. Moreover, we introduce the concept of a "rating gap" to assess rating fairness, revealing both undervaluation and overvaluation among countries. Notably, China’s sovereign rating was underestimated between 2009 and 2011 and overestimated between 2013 and 2016. These findings underscore the criticality of government vigilance in monitoring sovereign debt and credit ratings to navigate potential post-COVID-19 sovereign debt crises.

## 1. Introduction

The COVID-19 pandemic brought about various changes, and one significant consequence was the substantial increase in global public debt. This surge in debt is poised to be one of the pandemic’s most burdensome legacies. In this high-debt environment, debtors will grapple with the delicate task of balancing multiple constraints, akin to walking on a knife’s edge, and the financial system’s fragility will be heightened. Particular attention should be given to sovereign credit ratings among the challenges arising from mounting debt.

In this context, it is important to note that China’s SCR experienced a downgrade from Aa3/AA- to A1/A+ in 2017 by two leading credit rating agencies, Moody’s and Standard & Poor’s. It followed 2016’s "negative" outlook and was Moody’s second downgrade in China since 1988. Despite the limited impact on China’s bond, stock, and exchange markets, the downgrading may harm market perceptions of China’s economy, especially if the downgrading trend continues to cross *the critical point*, where it transcends from a mere investment-related concern to a speculative one. Such a juncture could yield a considerably amplified impact on the financial markets compared to other variations in credit ratings. The decrease in a country’s SCR also has implications for the cost of borrowing for the government, banks, and businesses.

The downgrade of China’s SCR was the result of a complex interplay between domestic and international factors. Domestically, China’s high public debt and leverage ratio were cited as contributing factors to the downgrade. Additionally, the profound and complex international environment at the time also played a significant role. The global economy has undergone significant changes since the outbreak of the US subprime crisis and the European debt crisis, leading to an increase in sovereign debt problems and defaults on debt in some developed countries. These events have had a significant impact not only on these countries’ economies but also on the global economy.

**Fig 1:**
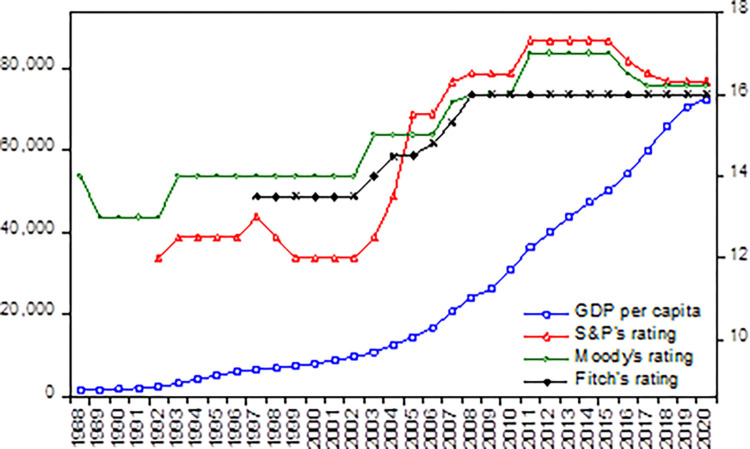
China’s sovereign credit rating and GDP per capital. Note: 1. This figure takes a linear numerical transformation of the rating letters and adjusts the positive/negative outlook to the 0.5 level. 2. The left axis of the figure is per capita GDP (unit: RMB), and the right axis is the rating values of the three leading agencies. 3. Source: World bank and Wind database.

Since Moody’s initial assignment of China’s sovereign credit rating in 1988, the two other leading credit rating agencies have also assigned credit ratings to China. Over the past three decades, China’s economy has undergone tremendous growth and development, as indicated by various economic indicators such as GDP per capita, total savings, total foreign exchange reserves, and total foreign debt. In turn, this growth and development have corresponded with a generally upward trend in SCR, with an improvement of four notches from an initial Baa1 to an Aa3 rating. The correlation between SCR and GDP per capita in China is depicted in Fig 1, which highlights the positive relationship between a strong economy and a higher SCR.

In the aftermath of the US subprime crisis, the economic indicators associated with China’s SCR have experienced a decline. Despite an overall increase in economic output, the growth rate has diminished, with GDP growth rates of 6.7% and 6.9% in 2016 and 2017, respectively, falling below the sustained 7% growth rate since 1991. Additionally, China’s foreign exchange reserves, the largest in the world for many years, have begun to drop, reaching $3.13 trillion at the end of 2017, a decrease of approximately $670 billion from its peak of $3.8 trillion. Furthermore, the fiscal deficit has increased yearly, surpassing the 3% threshold in 2015 and staying above that level in 2016 and 2017. These factors present significant challenges to China’s future SCR.

As countries embrace greater financial openness, the significance of sovereign ratings becomes increasingly apparent due to the rising demand for external financing. Furthermore, in the aftermath of the COVID-19 pandemic, concerns surrounding sovereign debt have become more prominent, highlighting the need to monitor and study sovereign credit ratings. The current characteristics of research in this field involve extensive data collection, the establishment of a scientific and accurate evaluation system, as well as the development of models, all of which cannot be accomplished overnight. Additionally, despite their significant impact, sovereign ratings do not directly generate economic income for rating agencies. These factors collectively define the nature of research in this field.

We conduct empirical analyses to examine the factors that influence SCRs and assess the impartiality of the three largest CRAs. Our research objective is to develop insights into how to mitigate the potential risks associated with a possible sovereign debt crisis in the future. The COVID-19 pandemic has resulted in a significant increase in government debt, which presents a challenge for the global economy in the post-pandemic era. As the governments withdraw supportive policies, the debt situation will deteriorate, making the financial system more fragile. In this context, it is imperative to pay close attention to SCRs, as they are a crucial indicator of a country’s creditworthiness. While the three leading CRAs did not widely downgrade SCRs during the pandemic [1], this may not continue.

This study makes fivefold contributions, with the first three being quantitative in nature, while the latter two are qualitative. Firstly, we enhance the conventional rating index system by introducing new indicators (outlined in Table 2), which are unique and not commonly used in other research papers. These additions, inspired by S&P’s rating system and incorporating qualitative factors, refine the evaluation framework. Secondly, utilizing the latest rating metrics, we categorize rating factors into short-term and long-term dimensions, building upon Alfonso’s framework. Our approach yields distinct findings and allows for targeted recommendations to address the debt crisis from both perspectives. Thirdly, we introduce a novel "rating gap" method to assess the impartiality of ratings, addressing a literature gap and contributing to the field of rating fairness. Moreover, it provides a basis for nations to implement measures safeguarding their rights. Fourthly, we assert the quasi-public goods attributes of sovereign credit ratings, a seldom discussed aspect. As these ratings at the national level do not directly generate income for rating agencies, smaller agencies hesitate to engage in this domain. Therefore, recognizing the long-term, public, and foundational nature of sovereign ratings, governments should increase financial support for scientific research in this field. Lastly, this study highlights the "critical point" effect of sovereign credit ratings. The critical point denotes the transition from an investment-grade to a speculative-grade level, which exerts a more significant impact on the financial market compared to other rating changes. Overall, these contributions enhance our understanding of sovereign credit ratings, provide practical recommendations, shed light on rating fairness, and highlight the quasi-public goods nature of SCRs.

The remainder of the paper is structured as follows: Section 2 provides a literature review; Section 3 describes the selection of indicators and the setting of the model (Methodologies); Section 4 reports empirical results; Section 5 evaluates and discusses the predictive power of the model; Section 6 investigates the rating fairness of China and related countries; Section 7 conducts the robustness checks; and Section 8 is the conclusion and policy implications.

## 2. Literature review and theoretical analysis

The existing literature on Sovereign Credit Ratings (SCRs) is abundant and encompasses a broad range of topics, including: (1) the determinants of SCRs; (2) the impacts of SCRs on macroeconomics and finance, particularly on bond, stock, exchange, and financial derivatives (e.g., CDS); (3) the fairness of SCRs; (4) the relationship between SCR and bank rating; (5) the impact of the "Sovereign ceilings" rule; (6) the comparative study of SCRs on EMEs versus developed countries; (7) the difference of rating methodologies of three leading CRAs; (8) the impact of political risks and uncertainty on ratings; (9) sovereign shadow ratings; (10) the relationship between SCRs and corporate strategic decisions. In this study, we have selected three relevant topics to be covered in the literature review.

### 2.1. The deterrent force of sovereign ratings

Due to information asymmetry and the requirement for knowledge when making investment and portfolio decisions on the global asset market, investors rely heavily on CRA reports [2,3]. The three leading CRAs (S&P, Moody’s, and Fitch) have a monopoly on the SCR industry, accounting for more than 95% of the market. SCR reports will influence investment behavior and market stability [4,5]. In most cases, bond prices are set by investors based on the bonds’ credit ratings [6,7]. Higher rated bonds typically have lower financing costs than lower rated bonds.

More importantly, SCR is a benchmark for a nation’s credit system, especially for the external financing rating, with *sovereign credit ceiling* effects. The concept of sovereign credit ceiling entails that the credit ratings assigned to all entities within a country, including banks, corporations, and local governments, are constrained by the sovereign credit rating. Consequently, any downgrade in the sovereign rating inevitably triggers cascading downgrades in the ratings of other entities, thus establishing a pervasive ceiling and benchmark for the entirety of the credit rating landscape [8–11]. In most cases, a local government, bank, or business can only have a lower credit rating than the SCR of that country. Consequently, the change of SCRs will affect bond, stock, and foreign exchange markets. A downgrade of a nation’s SCR may lead to a domino effect of negative events and impacts the creditworthiness and funding costs of all rated entities in the nation.

Despite never doing so before, in 1997, S&P announced it would begin to change its standards and give some firms in dollarized economies higher ratings than their respective sovereigns. Has this breached the sovereign ceiling norm and exceeded it on a large scale? Based on data from developed and emerging economies throughout 1995–2009, Borensztein and Cowan [9] discovered that SCRs continue to affect the company’s ratings, particularly for nations with strong capital controls and high political risks. The three major CRAs have only marginally violated this guideline. The SCR is still employed as a key benchmark and factor in establishing the enterprise’s rating. Additionally, the impact on financing costs is not equal when switching from low to high SCRs; for example, moving from the n-1st to the nth grade may not be similar to moving from the n-3rd to the n-2nd grade. Under the actual ratings, the *Critical Point effect* is typically triggered during the transition from Investment to Speculative Grade, which significantly impacts the market more than the general migration.

### 2.2. The fairness of sovereign ratings

Fairness is a critical aspect of determining a country’s sovereign creditworthiness. In recent years, concerns have been raised about the reliability of SCRs, with increasing public scrutiny regarding their impartiality and objectivity [12–14]. The immense impact of SCRs on financial markets has resulted in ongoing debates about their fairness [15,16]. When a rating agency announces a downgrade of a country, it can become a focal point of public opinion and face accusations and criticism from the downgraded nation. Furthermore, CRAs often cite protecting trade secrets as a reason for not disclosing their practices, exacerbating the issue. The downgrading of a country’s SCR is highly publicized and widely debated, attracting attention and scrutiny worldwide.

The dependability of CRAs in evaluating sovereign creditworthiness has been a subject of intense scrutiny. Some studies have pointed to the pro-cyclical nature of CRAs and inability to predict financial crises. For example, Ferri et al. [17] found that the CRAs not only failed to anticipate the onset of the East Asian financial crisis but also downgraded countries in its aftermath, exacerbating the situation. Host et al. [18] similarly explored how CRAs responded to the Eurozone debt crisis and concluded that they faced criticism for not giving market danger signals prior to the crisis. Using linear panel data and ordered probit methods, Fuchs [19] found that Moody’s and Fitch assign the United States a higher rating than it deserves. He also suggested the need for stronger oversight of SCR agencies to prevent the three leading agencies from monopolizing the market’s excess revenue. In contrast, Mora [20] argued that ratings are sticky, not pro-cyclical, and that CRAs provide independent assessments of a country’s likelihood of default. Kaminsky and Schmukler [21] emphasized that investors should not be overly reliant on these assessments and that the market should not be over-interpreted.

The results of sovereign credit rating processes have been a much-debated topic in the literature. However, a closer examination of the evidence suggests that the situation may be more complex than previously thought. Doluca [22] investigated the potential biases in the SCR process and found no significant evidence of *Home Country Bias* or *Profit Maximization* influencing the ratings. On the other hand, Reputation concerns were found to be a significant factor in the ratings, being more important than economic interests [23,24]. To maintain their reputation and credibility, Standard & Poor’s, Moody’s, and Fitch have declared that they will not engage in any form of manipulation that could affect a country’s economic growth, such as artificially raising or lowering a country’s credit rating [25]. This is further evidenced by Standard & Poor’s downgrade of the credit rating of the U.S., its home country, in 2011. However, there is ongoing debate as to whether these agencies follow scientific procedures in their work. For instance, it has been noted that the three agencies generally award higher scores to developed countries in their ratings [26]. This approach is understandable, as the solvency of high-income countries is generally more robust than that of low-income countries.

The ongoing disputes surrounding sovereign credit ratings highlight the need for an objective criterion in the rating process. To achieve this, it is important to understand the metrics and models utilized in determining SCRs. If this process is publicly disclosed, the fairness of SCRs will be comparatively solved, and it will be simple to determine whether countries are overstated or understated.

### 2.3. Determinants of sovereign rating

Transparency and disclosure of the factors used in determining sovereign credit ratings are crucial in reducing controversies and increasing the credibility of the ratings [13,27]. However, the models utilized by the three major CRAs are considered confidential information, leading to disparities in their ratings [28]. According to the indicators and ratings released by the three leading CRAs, scholars have found that the construction of a rating model primarily consists of several factors, including rating indicators, number of countries, measurement methodologies, and rating conversion methods, Etc. Despite the limited number of published indicators available for fitting the rating models, these factors are critical in determining the SCRs.

In the existing literature, Cantor and Packer [29] were pioneers in the study of sovereign credit ratings and proposed a method known as the CP model, which identified six significant criteria in determining SCRs. Subsequently, several academics have continued to improve the evaluation methods and procedures of SCRs from various perspectives: Firstly, the range of explanatory variables has been broadened. The six explanatory variables in the initial CP model were found to be limited, and some were insignificant in subsequent regression tests. Some studies [18,30] substituted the CP model’s total foreign debt indicator with the current account surplus and foreign exchange reserve, thereby improving the model’s goodness of fit. Other studies [31,32] have chosen different explanatory variables, but with the common trend of increased quantity and improved quality. To avoid collinearity and overlap, Mellios et al. [33] used principal component analysis (PCA) to optimize the explanatory variables with the addition of more variables to the equation.

Secondly, in the process of modeling credit ratings, the selection and preprocessing of dependent variables is crucial. To begin with, the credit rating notations, such as "A, B, C, D," and "+, -" symbols, must be transformed into numerical scales [34,35]. There are two primary conversion methods, linear and nonlinear. The linear method equalizes the intervals between each rating, while the nonlinear method does not [36,37]. Thirdly, econometric methods are refined and optimized. Given that the credit rating data is both cross-sectional and time-series, most studies utilize panel data regression techniques [38,39]. On the other hand, since credit rating results are expressed in an ordered discrete form, the multi-ordered selection model is a suitable alternative for this type of research.

In recent years, several new factors, including ESGs, machine learning, and artificial intelligence, have been incorporated into the field of sovereign credit ratings (SCRs), driving its development. The significance of Environmental, Social, and Governance (ESG) factors in determining sovereign credit ratings has been discussed. Pineau et al. [40] emphasize the importance of integrating ESG considerations into the assessment of countries’ creditworthiness. Similarly, Karaman [41] investigates the impact of countries’ ESG ratings on sovereign credit default swaps, offering empirical evidence from OECD countries between 2008 and 2019. The study highlights the influence of ESG ratings on credit risk and underscores the relevance of ESG factors in sovereign credit analysis. Overes and van der Wel [42] employ machine learning techniques to model sovereign credit ratings, assessing their accuracy and determining the driving factors behind these ratings.

In conclusion, while research in this field has continuously grown and evolved, deterrence, fairness, and determinants remain three interrelated and critical aspects of analyzing sovereign credit ratings. Firstly, due to the deterrent effect of SCRs on financial markets, market participants pay closer attention to the ratings and strive for their fairness. Secondly, to assess the objectivity of the rating system, it is essential to examine the factors that determine a nation’s credit rating. Improving the transparency of the rating process and objectively disclosing the variables that influence SCRs can mitigate disputes and enhance the credibility of the rating results.

Despite the extensive literature available on sovereign credit ratings, there are still several gaps in our current understanding of this field. These gaps encompass issues such as the fairness of sovereign credit ratings (SCRs), the inherent lag in these ratings, and the challenges posed by the quasi-public goods nature of SCRs. However, one primary challenge or bottleneck in sovereign ratings lies in the construction of the rating model. The moment the rating model is disclosed, the rating results gain more credibility. The impact and influence of sovereign ratings on financial markets have been extensively examined by researchers. However, these studies have significantly lagged the needs of financial practice, particularly in terms of adopting new theories and methodologies. Accurately revealing the rating model would be advantageous in protecting the interests of specific countries, uncovering the truth, and countering intentional distortions.

To address these gaps, this study aims to contribute to the literature by presenting novel and comprehensive indicators, as well as utilizing more accurate measurement techniques. The purpose of this study is to improve the existing literature on SCRs and provide policy recommendations in the context of the ongoing COVID-19 pandemic and its potential impact on triggering a global sovereign debt crisis.

## 3. Indicators selection and model setting

### 3.1. Dependent variables

The dependent variable in this study is the SCRs level assigned by credit rating agencies. The rating level is expressed as a combination of letters and symbols (e.g., "AAA/Aaa" and "+ -"), with AAA/Aaa indicating the highest rating and D representing the lowest default level. On average, CRAs assign more than 20 levels of ratings, with the majority falling in the moderate range. It is commonly recognized that ratings above "BBB-" are considered "investment grades," while those below "BBB-" are considered "speculative grades." Additionally, CRAs often place a country on a separate list when anticipating a change in its SCR. For instance, Standard & Poor’s classifies such positions as "Credit Watch" and assigns them a "positive," "negative," or "stable" rating, which is then officially published one to three years later.

In the realm of financial modeling, it is not feasible to model letter symbols as they are. Hence, a conversion process is necessary to represent these symbols in numerical form. This conversion can be accomplished through either objective or subjective techniques. In this study, we adopt a more objective linear transformation method, which assigns numerical values ranging from 20 to 1 to represent the credit ratings, with 20 being assigned to the highest rating of AAA/Aaa and 1 to the lowest rating of D (Table 1). This approach offers a more objective and systematic representation of credit ratings for financial modeling purposes.

**Table 1:** Classification and numerical transformation of SCRs.

Rating classification		Credit Rating Agencies			Rating Scale
		S&P	Moody’s	Fitch	
Investment grade	Prime/Highest quality	AAA	Aaa	AAA	20
	High quality	AA+	Aa1	AA+	19
		AA	Aa2	AA	18
		AA-	Aa3	AA-	17
	Upper Medium Strong payment capacity	A+	A1	A+	16
		A	A2	A	15
		A-	A3	A-	14
	Lower Medium Adequate payment capacity	BBB+	Baa1	BBB+	13
		BBB	Baa2	BBB	12
		BBB-	Baa3	BBB-	11
Speculative grade	Likely to fulfil obligations, ongoing uncertainty	BB+	Ba1	BB+	10
		BB	Ba2	BB	9
		BB-	Ba3	BB-	8
	High credit risk	B+	B1	B+	7
		B	B2	B	6
		B-	B3	B-	5
	Substantial/Very high credit risk	CCC+	Caa1	CCC+	4
		CCC	Caa2	CCC	3
		CCC-	Caa3	CCC-	2
	Near Default	CC/C	Ca/C	CC/C	1
	Selective Default/Default	SD/RD		D	0

### 3.2. Explanatory variables

The selection of explanatory variables for sovereign credit ratings in this study is guided by two perspectives: *ability* and *willingness*. A comprehensive literature review was conducted, encompassing seminal works such as Cantor and Packer [29] and Anfoson [43], as well as 51 indicators from Standard & Poor’s. Based on this, a screening methodology was developed, categorizing variables into six distinct categories that capture different aspects of sovereign ratings: macroeconomics, monetary borrowing, government balance, the balance of payments, external balance sheet, and central government debt and borrowing. By eliminating duplicate and missing data, 22 explanatory variables were identified as the most relevant, with 19 about debt repayment capacity and the remaining three focusing on willingness (detailed information in Table 2).

**Table 2:** Definitions of the explanatory variables.

Variable name	Definition	Sign
*1*.*Macroeconomic variables (6)*		
GDP per capita	Nominal GDP per capita (US$)	+
Real GDP growth	Real GDP growth rate (%)	+
Unemployment	Unemployment rate (%)	–
Inflation	GDP deflator growth (%)	+/–
Real investment growth	Real investment growth (%)	+
Savings/GDP	Savings/GDP (%)	+/–
*2*. *Government data (5)*		
GG balance/GDP	General government balance/GDP (%)	+
Net GG debt/GDP	Net general government debt/GDP (%)	-
LT commercial borrowing	Central GOV LT commercial borrowing	–
Commercial debt stock	Central GOV commercial debt stock	–
ST debt	Central GOV short term debt (US$ bil.)	–
*3*. *External Balance of payments (7)*		
Current account balance/GDP	Current account balance/GDP (%)	+
CARs/GDP	Current account revenues/GDP (%)	+
Usable reserves /GDP	Usable reserves /GDP	+
Narrow net ext. debt /CARS	Narrow net external debt /CAR_S_	–
ST ext. debt /CARs	Short term external debt /CARs	–
Usable reserves /CAPs	Usable reserves /CAPs	+
Net FDI/GDP	Net FDI/GDP	+
*4*. *Institutional variables (2)*		
Government effectiveness	Quality of public policy implementation	+
Regulatory Quality	Ability to formulate sound policies	+
*5*. *dummy variables (2)*		
Developed Economies	Taking a value of “1” for a developed country	+
Default History	Taking a value of “1” for a default country	-

Note: “+,–” in () is the expected direction of the parameter.

Our sample includes data from 73 nations and spans from 2009 to 2016, which covers the European debt crisis. The choice of this time period was driven by two factors: first, the European debt crisis represents the most recent severe sovereign debt crisis; and second, the observed parallels between the debt position during this period and the current global economic situation post-COVID-19. The data used in this study was obtained from the World Bank, IMF, and S&P databases.

In the credit rating process, the assessment of the borrower’s willingness to repay is of utmost importance, but it is often challenging to accurately gauge. Some previous studies have neglected the willingness aspect and focus solely on the ability to repay. However, it is widely acknowledged that not all entities with the capacity to repay their debts will fulfill their obligations. In the past, some governments have demonstrated the ability to repay but lacked the willingness to do so. To address this issue, our study employs three proxies for measuring repayment willingness: default history, government efficiency, and regulatory quality. Default history provides insight into an individual’s or government’s pattern of debt compliance and likelihood of defaulting in the future. Meanwhile, government effectiveness and regulatory quality serve as indicators of a nation’s level of civilization, legalization, and adherence to international standards.

### 3.3. Sovereign credit rating model

In this study, we employed two primary modeling approaches: panel linear regression and ordered probit regression. The linear regression model, being a conventional approach, is suitable for capturing pertinent information from both cross-sectional and time-series perspectives, given the inclusion of such data in sovereign credit ratings. Model selection among the three potential models was performed through F-tests and Hausmann tests. Additionally, considering that credit ratings are expressed in an ordered discrete form, ordered probability models prove suitable for this research. These models encompass the general ordered probability approach as well as the panel ordered probability approach. By utilizing ordered probability models, the determinants of sovereign ratings exhibit enhanced explanatory significance, overcoming the limitations of assuming equal rating distances in linear models and enabling estimation of the rating curve’s shape. However, it is important to note that ordered probability models possess certain drawbacks, including more demanding modeling conditions and potentially poorer regression results.

#### 3.3.1. Linear regression model

The panel linear regression model is a widely utilized method for estimating sovereign credit ratings. The basic form of the model is expressed as follows:

$$R_{it}=\beta X_{it}+\alpha_{i}+\mu_{it}$$ (1)

Where *i* and *t* are the subscripts for country and year, respectively. *R*_*it*_ denotes the transformation of rating variable, *X*_*it*_ is the vector of explanatory variables, and *α*_*i*_ represents the individual effect of country *i*. The disturbance term *μ*_*it*_ is further assumed to be independent of the cross-section and time-series variables. This model provides a basic framework for analyzing the relationship between the credit rating and a set of relevant explanatory variables. The estimated coefficients can be used to assess the impact of each explanatory variable on the credit rating. The implementation of this panel linear regression model will provide insights into the determinants of sovereign credit ratings.

Eq (1) can be estimated using three methods: pooled Ordinary Least Squares (OLS), fixed effects, or random effects. When *E*(*α*_*i*_|*X*_*it*_) = 0, it implies no correlation between the national effect and the explanatory variable, and thus the estimators for all three methods are equivalent. According to Afonso [43], the random effect model is the most efficient estimation technique, followed by the fixed and mixed effect models. However, in practice, the country effect *α*_*i*_ is often correlated with the explanatory variable *X*_*it*_, leading to inconsistent estimators when using the random or mixed effect models. As a result, the fixed effects model is often considered the most effective, as some researchers have demonstrated. Additionally, since the dependent variable (the credit rating) is typically sticky and changes little over time, the limited variation in the credit rating over time results in a lack of explanatory power.

Given the above concerns, we adopt Wooldridge’s [44] method as a solution. Our objective is to establish a clear association between the section error and explanatory variables. Wooldridge’s method provides a rigorous framework for addressing this issue, and has been widely accepted in the academic community as a reliable approach. The objective of this approach is to establish a robust relationship between the two variables.


$$E(\alpha_{i}|X_{it})=\eta {\bar{X}}_{i}$$ (2)

If we specifically change Eq (2) to $\alpha_{i}=\eta {\bar{X}}_{i}+\varepsilon_{i}$ and replace it into Eq (1), we obtain:

$$R_{it}=\beta X_{it}+\eta {\bar{X}}_{i}+\varepsilon_{i}+\mu_{it}$$ (3)

Where *ε*_*i*_ denotes the error term not related to the explanatory variable, the issue of prior correlation is addressed in this methodology by incorporating the mean of the explanatory variables into the regression equation. By modifying Eq (3), it can be expressed as:

$$R_{it}=\beta (X_{it}-{\bar{X}}_{i})+\delta {\bar{X}}_{i}+\varepsilon_{i}+\mu_{it}$$ (4)

The coefficients in Eq (4) provide insight into the factors affecting sovereign ratings. The term *δ*, defined as the sum of *η* and *β*, represents the overall impact of the rating on the long-term stability of the economy. The component *β* specifically highlights the short-term effects, offering important information for policymakers in formulating effective strategies to improve sovereign ratings. These distinctions provide a valuable tool for considering both short- and long-term perspectives in enhancing the stability of the economy.

#### 3.3.2. Ordered probit model

Given the commonly used representation of ratings as discrete, sequential letters (e. g. "A," "B," "C," and "D") with additional "+" and "-" symbols, the ordered probit model is well suited for rating modeling. CRAs often employ approximately 20 levels, with varying intervals between them, which meet the requirements of ordered probit modeling. Our study assumes that R* is a latent continuous variable that underlies the discrete dependent variables and has a linear relationship as follows:

$$R_{it}^{*}=\beta (X_{it}-{\bar{X}}_{i})+\delta {\bar{X}}_{i}+\varepsilon_{i}+\mu_{it}$$ (5)

The discreteness of the actual rating result *R*_*it*_ can be determined by dividing the continuous variable R* into sections, each of which corresponds to a different border range, and calculating the final rating as follows:

$$R_{it}=\left\{ \begin{array}{l} \;AAA(Aaa)\;if\;R_{it}^{*}>c_{17}\\ AA+(Aa1)\;if\;c_{16}<R_{it}^{*}<c_{17}\\ \;AA(Aa2)\;if\;c_{14}<R_{it}^{*}<c_{15}\\ \;\ldots \\ <CCC(Caa2)\;if\;R_{it}^{*}<c_{1} \end{array}\right.$$ (6)

In Eq (5) and (6), *β*、*δ* and the cutting points *c*_1_−*c*_17_ are parameters that must be estimated. The probability of each rating *R* is:

$$\left\{ \begin{array}{c} \mathrm{Pr}\left(R_{i}=1|X_{i},\beta_{i},\delta_{i},c\right)=\mathrm{Pr}\left(R_{i}^{*}\leq c_{1}\right)=\mathrm{Pr}\left(\beta (X-\overset{-}{X})+\delta \overset{-}{X}+\varepsilon +\mu \leq c_{1}\right)\\ =F(c_{1}-\beta (X_{i}-\overset{-}{X})-\delta \overset{-}{X}-\varepsilon )\\ \mathrm{Pr}\left(R_{i}=2|X_{i},\beta_{i},\delta_{i},c\right)=\mathrm{Pr}\left({c_{1}\leq R}_{i}^{*}\leq c_{2}\right)=\mathrm{Pr}\left(\begin{array}{c} c_{1}\leq \beta (X-\overset{-}{X})+\delta \overset{-}{X}\\ +\varepsilon +\mu \leq c_{2} \end{array}\right)\\ =F(c_{2}-\beta (X_{i}-\overset{-}{X})-\delta \overset{-}{X}-\varepsilon )-F(c_{1}-\beta (X_{i}-\overset{-}{X})-\delta \overset{-}{X}-\varepsilon )\\ \ldots \\ \mathrm{Pr}\left(R_{i}=18|X_{i},\beta_{i},\delta_{i},c\right)=\mathrm{Pr}\left({c_{17}\leq R}_{i}^{*}\right)=\mathrm{Pr}\left(c_{17}\leq \beta (X-\overset{-}{X})+\delta \overset{-}{X}+\varepsilon +\mu \right)\\ =1-F(c_{17}-\beta (X_{i}-\overset{-}{X})-\delta \overset{-}{X}-\varepsilon ) \end{array}\right.$$ (7)

Where *F* in Eq (7) is the residual term’s cumulative distribution function (CDF), and depending on how the residual term distribution differs, we can use the probit, logit, and extreme value estimating techniques.

## 4. Empirical results

The empirical analysis of this study will proceed in the following steps: (1) linear panel data regression models, including ordinary least squares (OLS) and random-effects models; (2) panel-ordered-probit models with random-effects and general-ordered-probit regression. To distinguish between the short- and long-term effects, the explanatory variables are divided into two categories: the offset of the variables from the mean, denoted by $X_{i}-{\bar{X}}_{i}$, which represents the short-term effects, and the mean of the explanatory variables, denoted by ${\bar{X}}_{i}$, which represents the long-term effects. Furthermore, based on the significance of the variables, each model will be further differentiated into a restricted form, which excludes non-significant variables, and an unrestricted form, which incorporates all explanatory variables.

### 4.1. Panel linear regression results

#### 4.1.1. The Pooled OLS model

Table 3 presents the mixed-effect model estimations. The unrestricted model (1) and restricted model (2) demonstrate strong explanatory power, as indicated by the *R*^2^ values of 0.97 and 0.94, respectively. In the unrestricted model (1), 31 out of 40 explanatory variables are found to be significant. Meanwhile, in the restricted model (2), 29 variables are significant, except for the short-term influence of per capita GDP. The coefficients of the short and long-term variables for the two models differ. Some variables display significant long-term effects, while their short-term effects are relatively insignificant. GDP per capita and real GDP growth exhibit a positive and significant impact on sovereign credit ratings in both the short and long run. This implies a direct association between higher levels of GDP per capita and stronger real GDP growth with elevated ratings. The underlying rationale for this relationship can be attributed to several factors. Firstly, a higher GDP per capita serves as an indicator of a country’s economic well-being and income level, reflecting its capacity to fulfill debt obligations. Secondly, stronger real GDP growth signifies a growing economy with increased revenue potential, reducing the likelihood of default. These favorable economic indicators enhance investor confidence and contribute to the assignment of higher ratings to countries demonstrating robust economic performance. Inflation is positively related to the rating in the short term but significantly lowers the rating in the long run. This finding aligns with economic theories suggesting that moderate inflation promotes growth, while prolonged high inflation is detrimental to the economy. In the short term, the positive correlation between inflation and the rating can be attributed to the beneficial effects of moderate inflation on economic activity, including stimulating demand and reducing the real burden of debt. However, in the long run, persistent high inflation has adverse consequences such as eroding purchasing power, distorting price signals, and undermining investor confidence. These factors contribute to a significant decline in the rating. This finding underscores the importance of maintaining inflation at moderate levels for long-term economic stability and creditworthiness.

**Table 3:** Panel data mixed effect regression results.

	Model (1)		Model (2)	
	Short-term	Long-term	Short-term	Long-term
GDP per capita	0.001** (1.98)	0.003*** (10.37)	0.001 (1.58)	0.002*** (15.73)
Real GDP growth	0.138*** (-3.08)	0.471*** (-7.36)	0.113*** (-8.24)	0.574*** (-6.00)
Unemployment	-0.497*** (-161.82)	-0.082*** (-3.66)	-0.565*** (-21.49)	-0.087*** (-3.16)
GDP deflator growth	0.015*** (2.76)	-0.137*** (-13.21)	0.016*** (7.73)	-0.138*** (-22.07)
Real investment growth	-0.011 (-1.03)	0.281*** (12.18)		0.313*** (7.74)
Savings/GDP	0.014 (0.47)	0.159*** (13.07)		0.166*** (8.65)
GG balance/GDP	0.241*** (46.86)	0.098* (1.62)	0.183*** (15.87)	0.092* (1.86)
Net GG debt/GDP	0.019*** (3.03)	-0.009*** (-6.32)	0.014*** (5.70)	-0.010*** (-9.23)
LT commercial borrowing	0.003*** (4.51)	-0.014*** (-5.98)	0.003*** (5.58)	-0.015*** (-7.51)
Commercial debt stock	0.000 (0.69)	0.003*** (6.39)		0.003*** (7.55)
ST debt	0.000*** (3.09)	0.000*** (-3.86)	0.000*** (4.64)	0.000*** (-5.20)
CARs/GDP	0.020 (0.96)	0.003 (1.69)		
Current account balance/GDP	-0.081 (-1.55)	-0.141*** (-4.76)		-0.161*** (-10.04)
Usable reserve/GDP	1.671*** (-2.73)	-1.437 (-1.52)	1.983*** (-3.81)	-1.072* (-1.67)
Narrow net ext. debt / CARs	-0.014*** (-8.33)	-0.001** (2.09)	-0.013*** (-11.50)	-0.002* (1.72)
ST ext. debt/CARs	-0.025** (7.54)	-0.001 (0.80)	-0.022*** (5.81)	
Usable reserve / CAPs	0.197*** (2.28)	0.135*** (4.61)	0.267*** (6.78)	0.146*** (8.57)
Net FDI/GDP	0.017*** (9.43)	-0.005 (-1.28)	0.008*** (3.53)	
Government effectiveness	0.46*** (4.59)		0.525*** (5.58)	
Regulatory quality	0.003 (0.95)		0.018*** (5.32)	
Developed economy	1.694*** (9.58)		1.529*** (19.74)	
Default history	-6.695*** (-34.51)		-6.708*** (-27.64)	
Constant	6.071*** (59.35)		6.097*** (64.57)	
*R* ^2^	0.941		0.973	
countries	73		73	
Observations	217		217	

Note: This table reports the estimation results using panel data mixed effect method, with column (1) and (2) representing unrestricted and restricted models, respectively. The t-statistic is in parentheses.

***, **, * denote significant at 1%, 5% and 10%, respectively.

In conclusion, the Pooled OLS model, which combines cross-sectional data with time series data, is a suitable method for situations with limited data. The model allows for the efficient utilization of different categories of data and helps overcome limitations in data availability, leading to improved results and analysis.

#### 4.1.2. Panel data random effect regression

Despite some advantages, the above basic mixed-effect regression does not allow further study of national effects. Therefore, we applied the Hausman test to the panel data to determine the suitability of the random effects model. The results of the Hausman test, as presented in Table 4, indicated that the null hypothesis was accepted, making the random effects model an appropriate choice for further analysis. Table 4 presents the results of the random effects model. The unrestricted model (1) has an *R*^2^ of 0.59, whereas the restricted model (2) has an *R*^2^ of 0.54, indicating that the restricted model’s explanatory power was reduced after the removal of insignificant variables. In the unrestricted model (1), 33 out of the 40 explanatory variables were significant, whereas in the restricted model (2), all variables were significant. The results show that GDP per capita has a small and close to zero coefficient, suggesting that its impact on credit rating has weakened. The coefficient for GDP growth is positive, indicating that an increase in GDP growth by 1% leads to an increase in the credit rating by 0.47 points in the long run. The coefficient for the unemployment rate is negative, suggesting that a higher unemployment rate is detrimental to the credit rating. Additionally, the long- and short-term effects of inflation on credit rating are distinct. In the short term, inflation has a positive effect on credit rating, but in the long term, it has a negative effect. This is consistent with the economic viewpoint that moderate inflation can benefit economic growth, while consistently high inflation is detrimental.

**Table 4:** Panel data random effect regression results.

	Model (1)		Model (2)	
	Short-term	Long-term	Short-term	Long-term
GDP per capita	0.000*** (3.16)	0.002*** (4.10)	0.000*** (3.44)	0.001*** (12.95)
Real GDP growth	0.008 (0.30)	0.656** (-2.21)		0.471*** (-2.73)
Unemployment	-0.128*** (-14.84)	-0.067 (-1.14)	-0.164*** (-4.51)	
Inflation	0.004* (1.91)	-0.136*** (-4.39)		-0.125*** (-4.60)
Real investment growth	-0.010*** (-3.57)	0.394*** (5.89)	0.007*** (-2.67)	0.395*** (3.08)
Savings/GDP	0.051 (0.94)	0.190*** (5.41)		0.185*** (7.10)
GG balance/GDP	0.081*** (4.09)	0.005 (0.03)	0.070*** (18.19)	
Net GG debt/GDP	0.019*** (3.03)	-0.009*** (-6.32)	0.014*** (5.70)	-0.010*** (-9.23)
LT commercial borrowing	0.000 (0.82)	-0.018*** (-8.36)		-0.013** (-2.07)
Commercial debt stock	0.000 (0.31)	0.003*** (10.48)		0.003** (2.24)
ST debt	0.000*** (-4.10)	0.000*** (-2.92)		0.000*** (-10.73)
CARs/GDP	0.010 (0.88)	-0.002* (-1.80)		-0.004** (-2.38)
Current account balance/GDP	-0.045 (-0.63)	-0.128*** (-4.02)		
Usable reserve/GDP	-0.357 (-0.32)	1.769 (0.72)		
Narrow net ext. debt / CARs	0.000 (0.00)	0.001 (0.42)		
St ext. debt /CARs	0.001 (0.10)	-0.002 (-0.37)		
Usable reserve / CAPs	-0.029 (-0.38)	0.039 (1.39)		
Net FDI/GDP	0.001 (0.26)	-0.005*** (-2.69)		
Government effectiveness	0.51*** (6.56)		0.631*** (6.51)	
Regulatory quality	0.004 (0.86)		0.021*** (5.86)	
Developed economy	0.225*** (2.70)		0.175* (1.96)	
Default history	-1.492** (-2.48)		-1.343*** (-3.23)	
Constant	5.578*** (7.13)		4.959*** (8.43)	
*R* ^2^	0.586		0.538	
Countries	73		73	
Observations	217		217	
Hausman test	0.006 (0.98)		0.005 (0.99)	

Note: This table reports the estimation results using panel data random effect method, with column (1) and (2) representing unrestricted and restricted models, respectively. The Hausman test lists the chi-square values and the corresponding probabilities. The t-statistic is in parentheses.

***, **, * denote significant at 1%, 5% and 10%, respectively.

### 4.2. Ordered probit regression results

The ordered probit model overcomes the linear model’s assumption of equalization of the rating distance and estimates the rating curve’s form, providing additional explanatory implications for the factors influencing SCRs.

#### 4.2.1. General ordered probit model

According to our findings (see Table 5), the ordered-probit model has more significant explanatory variables than the mixed-effect model, and some of the variables’ symbols and coefficients are different from previous model. Some macroeconomic indicators, such as investment and savings, do not significantly impact the rating. In all government expenditure and debt indicators, only the government balance has a short-term impact, while the remaining variables have a substantial long-term impact on ratings. The useable reserve/GDP and narrow net external debt/CARs are insignificant among the external income and spending indicators. Whether or not a country is a developed economy has no significant effect on ratings, suggesting that the influence of this indicator is diminishing and confirming the results of earlier models.

**Table 5:** General ordered probit regression results.

**Panel A:**	**Model (1)**		**Model (2)**	
	**Short-term**	**Long-term**	**Short-term**	**Long-term**
GDP per capita	0.001*** (-2.29)	0.003*** (7.14)	0.001* (-1.78)	0.002*** (9.19)
Real GDP growth	0.112** (-2.02)	0.117 (0.87)	0.140*** (-2.91)	
Unemployment	-0.186*** (-3.03)	-0.044* (-1.77)	-0.194*** (-3.23)	
Inflation	0.055*** (-3.24)	-0.092*** (-5.90)	0.057*** (-3.27)	-0.099*** (-6.44)
Real investment growth	-0.015 (-0.87)	0.060** (2.25)		
Savings/GDP	0.020 (0.27)	0.044*** (2.58)		0.072*** (5.91)
GG balance/GDP	0.240*** (4.87)	0.106** (2.02)	0.235*** (6.31)	0.082* (1.83)
Net GG debt/GDP	0.029*** (2.74)	-0.008** (-2.08)		-0.008** (-2.16)
LT commercial borrowing	0.001 (0.29)	-0.013*** (-3.48)		-0.017*** (-5.04)
Commercial debt stock	0.002** (2.06)	0.003*** (3.50)		0.003*** (4.99)
ST debt	0.000 (-1.33)	0.000*** (-3.04)		0.000* (-1.81)
Current account balance/GDP	0.042* (1.71)	0.009** (2.39)		0.007*** (4.10)
CARs/GDP	0.106** (-2.00)	-0.082** (-2.42)	0.083*** (-2.94)	-0.122*** (-5.30)
Usable reserve/GDP	0.117 (0.03)	-0.878 (-0.75)		
Narrow net ext. debt / CARs	-0.009** (-2.17)	0.001 (0.68)		
ST external debt /CARs	0.009* (1.83)	-0.003* (-1.61)		-0.004*** (-3.59)
Usable reserve / CAPs	0.134 (1.34)	0.086* (1.88)		0.076*** (3.17)
Net FDI/GDP	0.024*** (3.72)	-0.005 (-0.46)	0.018*** (5.38)	
Government effectiveness	0.60*** (5.13)		0.635*** (5.58)	
Regulatory quality	0.005 (0.65)		0.028*** (6.02)	
Developed economy	0.261 (0.78)		0.221 (0.69)	
Default history	-9.932*** -(17.91)		-9.663*** (-21.20)	
Log likelihood ratio	-376.53		-390.94	
Countries	73		73	
Observations	217		217	

**Panel B**: **Cutoff point**											
**Model (1)**						**Model (2)**					
Cut1	-2.60	Cut7	0.94	Cut13	3.85	Cut1	-1.60	Cut7	1.44	Cut13	4.26
Cut2	-1.69	Cut8	1.34	Cut14	4.63	Cut2	-0.78	Cut8	1.84	Cut14	4.99
Cut3	-0.76	Cut9	2.02	Cut15	5.10	Cut3	-0.03	Cut9	2.51	Cut15	5.42
Cut4	-0.13	Cut10	2.66	Cut16	5.48	Cut4	0.48	Cut10	3.12	Cut16	5.74
Cut5	0.16	Cut11	3.11	Cut17	5.91	Cut5	0.73	Cut11	3.55	Cut17	6.12
Cut6	0.55	Cut12	3.76			Cut6	1.06	Cut12	4.17		

Note: This table reports the estimation results using general ordered probit method, with column (1) and (2) representing unrestricted and restricted models, respectively. The t-statistic is in parentheses.

***, **, * denote significant at 1%, 5% and 10%, respectively.

Panel B of Table 5 provides an estimation of 17 cutoff points for the model. The distance between the cutoffs is not uniform, and notably, there is a significant increase in the threshold between the BBB- and BBB ratings. The distance between the levels varies, with a decrease and subsequent increase in the portion above the investment level, suggesting that achieving a higher rating is increasingly difficult.

#### 4.2.2. Panel order probit model

Table 6 presents the results of the panel ordered probit model. Compared to the random effects model discussed in Section 4.1.2, this model includes three additional insignificant explanatory variables and some differences in symbols and coefficients. Our results show that the variables of investment, GDP growth rate, long-term unemployment, and short-term savings do not have a significant impact on ratings. Regarding the impact of government spending and debt variables on sovereign ratings, we find that a balanced budget and a medium debt/GDP ratio are favorable for sovereign ratings, as reflected in the sign and significance of the long-run coefficients of the variables in the model. This suggests that countries with disciplined fiscal policies, characterized by a balanced budget and a manageable debt-to-GDP ratio, are more likely to receive higher ratings. A balanced budget signifies responsible financial management, where a country’s revenue matches its expenditure. Similarly, a manageable debt-to-GDP ratio implies that the country’s debt burden is sustainable and not excessively burdensome. These factors contribute to higher sovereign ratings as they reflect stability and the ability to meet financial obligations. Overall, the findings highlight the importance of maintaining fiscal discipline in order to enhance a country’s rating in the sovereign market. The variables of current account income/GDP, narrow net external debt/CARs, and short-term external debt by remaining maturity/CARs have a short-term effect on ratings. These variables are crucial in capturing key aspects of a country’s external position. A surplus in the current account signifies a robust economic performance and the ability to meet external obligations, as it indicates that a country is earning more from exports and investments than it is spending on imports and interest payments. Furthermore, a lower level of narrow net external debt relative to CARs indicates reduced vulnerability to external shocks and a lower likelihood of default. Similarly, a reduced reliance on short-term debt suggests a decreased risk of liquidity pressures. Consequently, maintaining a favorable external position characterized by a surplus in the current account, an adequate level of foreign reserves, and a diminished reliance on short-term debt is beneficial for sovereign credit ratings. We also find that the country’s status as a developed economy remains insignificant, while the history of default has a higher negative impact on ratings. On the other hand, government efficiency and regulatory quality have a significant positive correlation with the rating.

**Table 6:** Panel random effect ordered probit regression results.

**Panel A:**	**Model (1)**		**Model (2)**	
	**Short-term**	**Long-term**	**Short-term**	**Long-term**
GDP per capita	0.003*** (4.82)	0.005*** (6.83)	0.001*** (5.34)	0.002*** (8.94)
Real GDP growth	-0.014 (-0.59)	-0.562 (-1.11)		
Unemployment	-0.343*** (-9.06)	-0.082 (-0.79)	-0.350*** (-9.76)	
Inflation	-0.065*** (-5.57)	-0.132*** (-3.18)	0.147*** (-3.86)	-0.064*** (-5.86)
Real investment growth	0.001 (0.16)	0.351 (1.51)		
Savings/GDP	0.028 (0.85)	0.208*** (2.51)		0.181*** (4.07)
GG balance/GDP	0.027 (1.30)	0.139 (0.74)		
Net GG debt/GDP	-0.047*** (-7.88)	-0.009 (-0.56)	-0.047*** (-8.70)	
CARs/GDP	0.017*** (1.69)	0.00 (0.60)	0.017** (2.00)	
Current account balance/GDP	-0.034 (-1.36)	-0.211* (-1.74)		
Usable reserves/GDP	0.414 (0.23)	2.167 (0.51)		
Narrow net ext. debt/ CARs	-0.008*** (3.74)	0.010 (1.45)	-0.008*** (3.61)	
ST external debt /CARs	-0.006*** (3.68)	-0.002 (-0.45)	-0.006*** (3.67)	
Usable reserves/CAPs	0.062 (1.07)	0.093 (0.51)		
Net FDI/GDP	0.006 (0.64)	-0.039 (-0.82)		
Government effectiveness	0.56*** (4.80)		0.671*** (6.10)	
Regulatory quality	0.006 (1.15)		0.030*** (6.19)	
Developed economy	-0.125 (-0.29)		-0.118 (-0.19)	
Default history	-2.031*** (-3.43)		-1.926*** (-3.28)	
Log likelihood ratio	-807.9		-816.4	
Countries	75		75	
Observations	600		600	

**Panel B: Cutoff point**							
**Model (1)**				**Model (2)**			
Cut1	-1.74	Cut10	7.70	Cut1	-1.97	Cut10	7.37
Cut2	-0.82	Cut11	8.48	Cut2	-1.05	Cut11	8.16
Cut3	0.21	Cut12	9.47	Cut3	-0.03	Cut12	9.14
Cut4	1.17	Cut13	9.92	Cut4	0.89	Cut13	9.58
Cut5	2.27	Cut14	11.30	Cut5	1.97	Cut14	10.94
Cut6	2.97	Cut15	12.60	Cut6	2.67	Cut15	12.23
Cut7	3.86	Cut16	14.02	Cut7	3.55	Cut16	13.63
Cut8	4.99	Cut17	15.34	Cut8	4.68	Cut17	14.94
Cut9	6.29			Cut9	5.96		

Note: This table reports the estimation results using panel random effect ordered probit method, with column (1) and (2) representing unrestricted and restricted models, respectively. The t-statistics are in parentheses.

***, **, * denote significant at 1%, 5% and 10%, respectively.

Panel B of Table 6 provides an estimation of 17 cut points. Our results indicate that the distance between the levels is shorter at lower ratings but increases as the rating improves, suggesting that achieving a higher rating becomes increasingly challenging.

## 5. Evaluation and discussion of model predictive power

### 5.1. Evaluation of model predictive power

In line with previous literature [44,45], we assess the predictive power of several models by comparing the fitted to the actual values. We utilize the estimated coefficients obtained in Section 4 and substitute each country’s data into the relevant model, as illustrated in Table 7. In addition to the four types of eight models discussed earlier, this table includes nine models, including the differentiation of panel random-effects models into two models, one with and one without the national effect (*V*_*i*_).

**Table 7:** Summary of model prediction error.

Model type	Obs.	Prediction error (number of notches)					%Correctly predicted	%Within1 notch	%Within 2 notches
		-2	-1	0	1	2			
Unres. Pooled OLS	217	16	23	91	25	26	41.9	64.1	83.4
Res. Pooled OLS	217	21	17	91	28	22	40.1	62.7	82.5
Unres. Random Effect	217	25	23	61	29	26	48.1	72.1	85.5
Res. Random Effect (without *V*_*i*_)	217	25	24	59	26	32	47.2	70.2	81.5
Res. Random Effect (with *V*_*i*_)	217	0	4	205	6	1	81.1	89.4	92.2
Unres. Ordered Probit	600	1	71	443	59	25	73.8	87.2	89.8
Res. Ordered Probit	600	0	82	441	52	22	73.5	85.8	88.5
Unres. RE Ordered Probit	632	13	25	64	68	80	82.2	93.9	96.8
Res. RE Ordered Probit	632	16	14	59	68	65	80.9	92.5	95.6

Note: Unres. represents a model with all explanatory variables, while Res. includes only significant ones. The number of notches refers to the difference between the predicted and actual value. V*i* refers to the cross-section effect.

Table 7 displays the results of an assessment of the predictive power of several models. The panel RE-ordered probit model has the highest prediction accuracy, at 82.2%. When allowing for a single error notch, the accuracy of the prediction increases to 93.9%. The panel random-effect model with the national effect *V*_*i*_ has the second-highest predictive power, with an accuracy of 81.1%. With the allowance for a single error notch, the accuracy of prediction can reach 89.4%. In comparison, the model with the weakest predictive performance is the Pooled OLS, with an accuracy rate of approximately 40%.

### 5.2. Discussion

The existing literature on the predictiveness of econometric models presents a mixed picture. Afonso [43] compares the panel random-effects model with the ordered probit model and finds that the former exhibits superior predictive potential, with an accuracy rate of 70% compared to the latter’s 35%. Conversely, Erdem and Varli [45] report that the panel mixed-effect model was the most predictive among the five models they evaluated, achieving a 61% correct rate within a rating notch. Our empirical investigation, however, reveals that the panel mixed-effect model has a lower correct rate of 42%, which diverges from the results obtained in previous studies.

The conflicting views in the literature on model predictiveness could be due to two potential causes. Firstly, it could be attributed to the characteristics of the explanatory variables. The rating model typically employs data from over 100 nations, incorporating more than 10 explanatory factors and 10 years of data. Our paper includes more than 20 explanatory variables, which increases the likelihood of complex calculation errors in the presence of missing data [45]. Secondly, the conflicting views could be related to the dependent variables, given that the rating model has many classified variables, typically over 16 categories and 20 rating levels. When there are too many categories, the coefficients calculated using the panel-ordered probit approach may need to be more accurate, and using a Binary Choice model may enhance the predictive power if the number of classifications is reduced to consolidate all categories into investment and speculative level categories. However, this would result in significant information loss.

## 6. Exploring rating fairness: China and related countries

In this section, we aim to evaluate the accuracy of ratings assigned to China and other similar nations. To accomplish this, we will conduct a SCR fitting and predictive analysis utilizing the panel RE-ordered probit model with maximum predictive power, as previously determined through a predictive power assessment (as shown in Table 7). To quantify the discrepancy between observed and predicted values, we introduce the concept of "rating gap," which is calculated as the difference between the observed and predicted values. The formula for the rating gap is provided as follows:

$${\Delta Ratings}_{i}={Ratings}_{act,i}-{Ratings}_{fit,i}$$ (8)

Where ΔRatings_*i*_ refers to the rating gap, *Ratings*_*act*,*i*_ represents the actual rating values, and *Ratings*_*fit*,*i*_ denotes the model fitted values. A positive rating gap indicates that the actual values are higher than the fitted values, implying a potential for overestimation. Conversely, a negative rating gap suggests that the actual values are lower than the fitted values, indicating a possibility for underestimation. This conclusion is supported by the static and trailing nature of static credit ratings, which are updated gradually in response to changing economic conditions. It is therefore important to consider the potential impact of the rating gap in evaluating credit risk.

We selected three distinct groupings of nations for the purpose of forecasting Sovereign Credit Ratings:

1. Greater China, which encompasses the mainland of China, Hong Kong, and Taiwan.
2. The G7, a representative group of developed nations consisting of Canada, France, Germany, Italy, Japan, the United Kingdom, and the United States. The aggregate Gross Domestic Product (GDP) of these nations accounts for approximately two-thirds of the world economy.
3. BRICS, consisting of Brazil, China, Russia, India, and South Africa, representing emerging market economies.

The selection of these three groupings provides a comprehensive representation of various economic conditions, ranging from developed to emerging markets. This allows for a thorough examination of the differing factors that influence the Sovereign Credit Ratings within each grouping.

**Fig 2:**
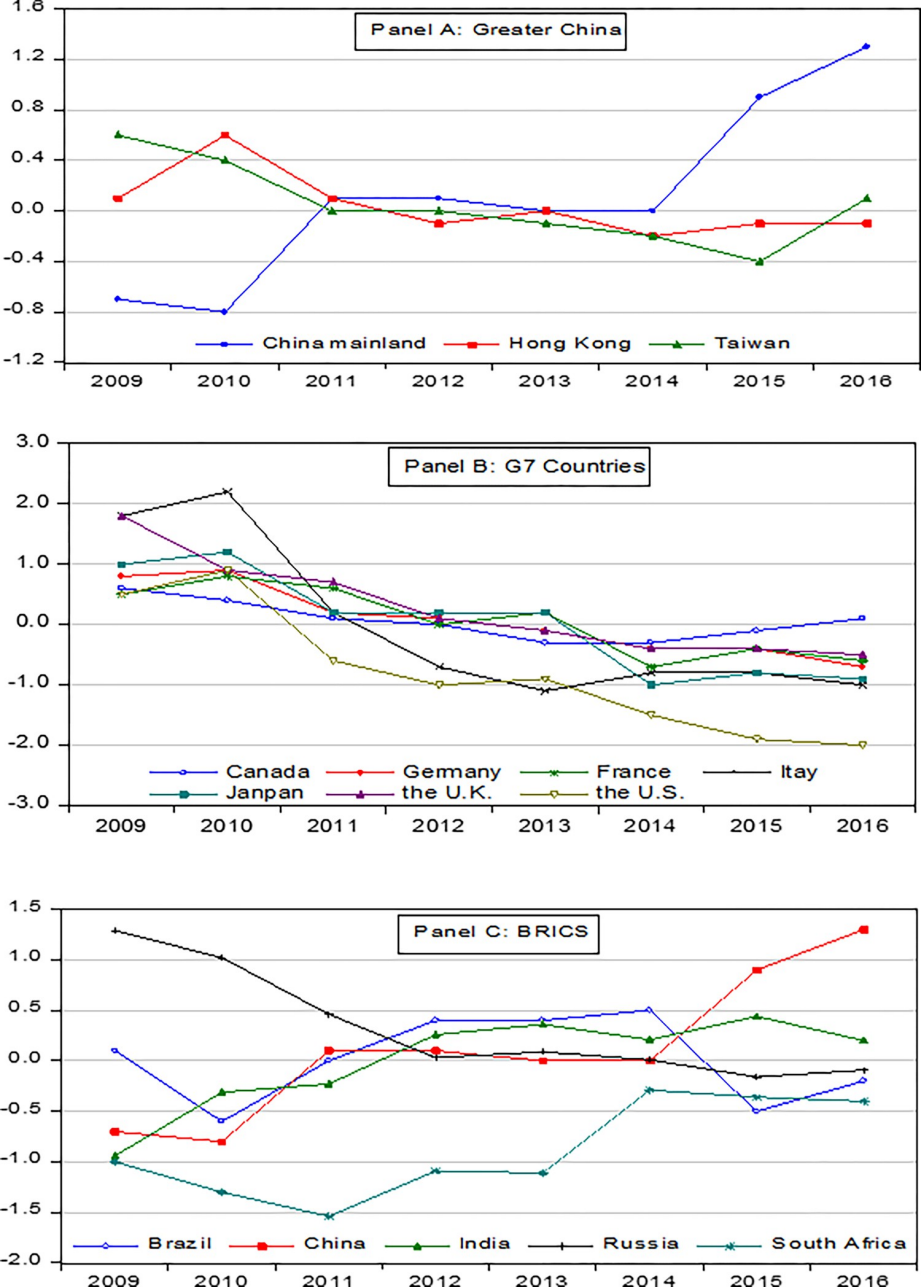
The actual and fitted rating gap for Greater China, BRICS, and G7 countries.

In the Panel A of Fig 2, the forecast for the Standard and Poor’s Credit Rating (SCR) in Greater China is demonstrated. Our analysis shows that there was a substantial difference in the SCR between 2009 and 2011 in mainland China. The calculated rating gap was approximately -0.8, implying that the SCR was underestimated by almost one notch during that time period. However, the situation improved from 2013 to 2016 as the rating gap shifted from negative to positive, reaching a peak of 1.3 points in 2016. This may suggest that the SCR was overestimated by one to two notches at this stage. In contrast, the credit rating of Taiwan showed a positive trend until 2015 but has since decreased, which can be attributed to the economic slowdown during those years. The credit rating of Hong Kong, on the other hand, remained constant despite significant changes in its socio-economic environment in recent years. It is worth noting that credit ratings are generally inflexible, and rating agencies provide outlooks before making revisions. If Hong Kong’s economy does not improve, the credit rating is likely to be modified.

Panel B of Fig 2 displays the trend in Standard and Poor’s Credit Ratings (SCRs) for the G7 developed countries. Our findings show that the SCRs of the U.S., the U.K., France, and Italy increased over the study period, despite the impact of the subprime mortgage crisis and the European debt crisis. This can be attributed to the improvement in their economies, leading to a tendency for the fitted values of the SCRs to surpass their actual values. Except for Canada, the remaining six countries in the G7 group showed a negative rating gap that continued to widen, with the highest negative gap being observed in the U.S. This suggests that the U.S. may return to a higher SCR in the future. Panel C of Fig 2 presents the SCRs of Emerging Market Economies (EMEs), represented by the BRICS countries. The results indicate that the SCRs of the BRICS countries are relatively stable and have only declined slightly. The SCR of Russia showed a downward trend, with both the actual and fitted values trending downward. India’s trend was mildly ascending and relatively stable, whereas Brazil and South Africa exhibited modest rating gaps, indicating that the actual and predicted values were close.

## 7. Robustness test

Robustness tests were conducted to enhance the reliability and validity of the findings, as mentioned above, across diverse settings. The study employed several approaches to ensure robustness. Firstly, this research focused on the top 20 developing countries globally, aiming to demonstrate the robustness of the findings across a broad range of study subjects. The selection of countries was based on the Standard & Poor’s Top-20 Emerging Markets (by outstanding debt) list, comprising 18 countries. From this list, four countries, namely Brazil, China, India, and South Africa, which were previously examined in Section 6, were excluded. This resulted in a final set of 14 countries for analysis. The countries included in the sample for the robustness test are Mexico, Indonesia, Saudi Arabia, Türkiye, Poland, Argentina, Thailand, Malaysia, Philippines, Egypt, Colombia, Qatar, and Hungary. Notably, all these 14 countries, except for Pakistan, which holds a CCC+ rating, possess speculative-grade ratings of BBB- or higher, indicating a relatively favorable credit standing. For further information, the official website for Standard & Poor’s ratings can be accessed at https://disclosure.spglobal.com/sri/.Secondly, the study period was adjusted to span from 2017 to 2021, thereby corroborating the temporal robustness of the results. Lastly, methodological improvements were implemented, including adopting a first-order lagged form for the explanatory variables. This modification aimed to mitigate endogeneity issues arising from reverse causality and address concerns regarding rating lag.

**Fig 3:**
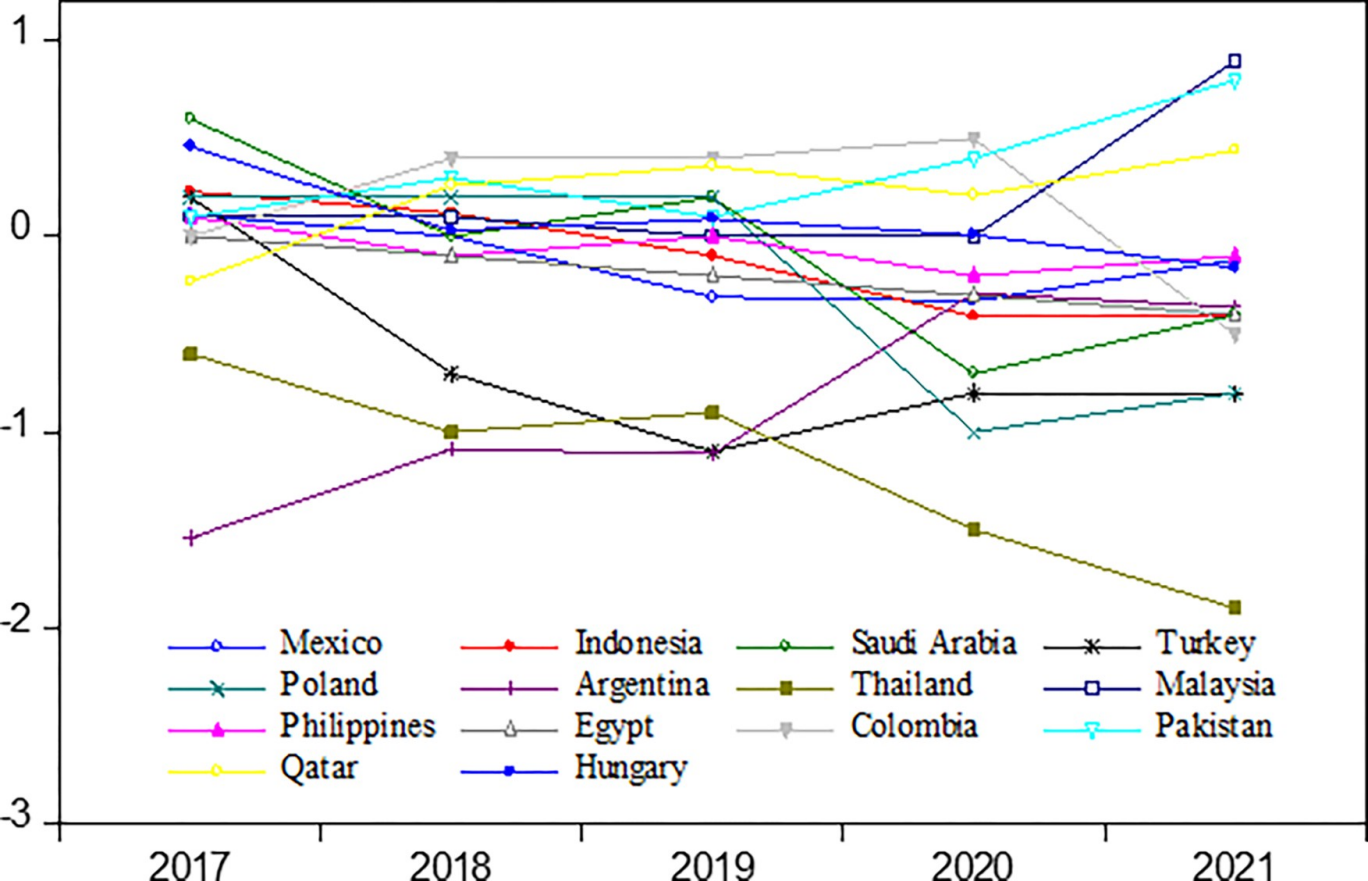
The actual and fitted rating gap for 14-EM countries.

Furthermore, this study examines the fairness of ratings for the 14 countries mentioned above, as illustrated in Fig 3. Due to the distinct circumstances of these countries, they exhibit individual characteristics with a need for commonalities. Certain countries, such as Turkey and Thailand, demonstrate significant fluctuations. Additionally, with the onset of the COVID-19 pandemic in 2020, the overall ratings of these countries tend to be overestimated.

The robustness test results, as presented in Table 8 and Fig 3, exhibit consistency with the baseline results in terms of the magnitude of coefficients and the signs of key explanatory variables. This evidence supports the robustness and consistency of the conclusions drawn from this study, from various perspectives such as across different time periods, samples, and methodologies.

**Table 8:** Panel random effect ordered probit regression results.

	(1) Panel random-effect		(2) Panel RE ordered probit	
	Short-term	Long-term	Short-term	Long-term
Log GDP per capita	0.002*** (3.16)	0.032*** (4.10)	0.003*** (4.83)	0.065*** (6.85)
Real GDP growth	0.009 (0.29)	0.689** (-2.19)	0.015** (-2.16)	0.483 *** (-8.01)
Inflation	0.005* (1.89)	-0.129*** (-4.51)	0.058* (-1.27)	-0.161*** (-3.18)
GG balance/GDP	0.071*** (4.21)	0.006 (0.02)	0.019 (1.30)	0.151*** (6.74)
LT commercial borrowing	-0.002 (0.85)	-0.019*** (-9.10)	-0.002 (0.23)	-0.017*** (-5.04)
Central GOV ST debt	-0.025*** (-5.10)	0.003 (-0.92)	-0.046*** (4.68)	0.002 (-0.45)
Current account balance/GDP	0.046 (-0.66)	0.129*** (-4.31)	0.034 (-1.36)	0.211* (-1.78)
Usable reserve/GDP	-0.356 (-0.31)	1.768 (0.69)	0.414 (0.23)	2.167 *** (6.51)
ST external debt /CARs	-0.001*** (6.10)	-0.002 (-0.37)	-0.006*** (3.66)	-0.002 (-0.44)
Government effectiveness	0.55*** (6.89)	0.603*** (6.58)	0.58*** (4.80)	0.671*** (6.13)
Regulatory quality	0.008 (0.83)	0.032*** (5.83)	0.006 (1.16)	0.030*** (6.15)
Default history	-6.663*** (-19.20)		-8.013*** (-21.21)	
R-squared	0.81		0.85	
Countries	14		14	
Observations	70		70	

Note: This table reports the estimation results using two methods, where each model is divided into long-term and short-term effects. The t-statistic is in parentheses.

***, **, * denote significant at 1%, 5% and 10%, respectively.

## 8. Conclusion and policy implications

The international community is concerned about the impartiality and determinants of sovereign credit ratings. we developed a new index system, divided the rating variables into long-term and short-term factors, performed rating fitting and prediction, and investigated the fairness of China and relevant countries.

Our findings are summarized as follows: Firstly, in the aftermath of the COVID-19 pandemic, the SCR indicator system has become increasingly crucial in adapting to the evolving global economic landscape. As such, we have sought to enhance the traditional SCR indicator system by incorporating a variety of new explanatory variables. This undertaking has been informed by the latest academic research and S&P rating indicators, resulting in the development of 22 comprehensive index systems covering macroeconomics, sovereign debt, and the international economy. Our efforts have refined and improved the rating model, enhancing its effectiveness and accuracy. Secondly, from the policymaker’s perspective, we separate the determinants of SCRs into long-term and short-term factors. Our research discovered that economic growth, unemployment, inflation, savings, government effectiveness, regulatory quality, and default history have long-term effects on ratings. In contrast, government revenue and expenditure and external debt have short-term effects on ratings. Thirdly, we proposed the "rating gap" as a means of evaluating rating fairness, with a particular focus on the G7, BRICS, and Greater China regions. Our analysis shows that China’s sovereign credit rating was undervalued by nearly one notch from 2009 to 2011 and overvalued by one to two notches from 2013 to 2016. In contrast, the G7 countries exhibited relatively strong ratings after 2013, with the United States’ performance being particularly noteworthy. Russia’s position among the BRICS nations has seen a decline, while India’s rating has remained relatively stable with a slight upward trend. The rating gaps in Brazil and South Africa are modest, with no significant deviation from the actual values.

Based on the above main research conclusions, the following policy recommendations were put forward: Firstly, in response to a sovereign debt crisis, we offer the government short- and long-term recommendations. To fundamentally enhance the sovereign credit rating and maintain a high rating in the long run, the country must prioritize improvement in the long-term determinants of its financial operations, such as increasing fiscal balance and foreign trade surplus, thus fortifying the overall solvency of the nation. In the short term, the government must also adopt effective measures for emergency debt management, including debt reduction, repayment, and swapping. Secondly, given the ceiling effect and indirect profitability, sovereign credit ratings exhibit characteristics of quasi-public goods. As a result, small to medium-sized rating firms are often reluctant to undertake national-level ratings, as they do not typically yield clear profits. However, as a country’s financial openness expands, its dependence on external funding also increases, elevating the significance of its sovereign credit rating. Furthermore, conducting a sovereign credit rating project necessitates collecting a substantial amount of data and developing an exact evaluation model, which cannot be achieved rapidly. Given the long-term, fundamental, and public nature of sovereign credit ratings, governments should offer increased financial and policy support for related research initiatives. Thirdly, emerging market economies have faced challenges in their development of credit ratings. The process started relatively late and has progressed slowly, leaving many domestic firms that finance abroad in these nations vulnerable to the ratings assigned by the three largest CRAs. Many EMEs, including China, must establish and enhance their SCR agencies and research organizations to address this challenge. This will increase their rating discourse power, strengthen their presence in international markets, and resist potential manipulation by international oligopolies. Furthermore, domestic rating agencies require a greater voice in international markets and a change in international rating agency legislation. Lastly, the COVID-19 pandemic has brought about significant shifts in the global political economy, presenting the potential for adverse conditions in sovereign debt. Governments should engage in ongoing monitoring and early warning strategies to mitigate these risks. EMEs must consider modifying the currency structure of their loans and claims and diversifying their reserve moderately. Concerning China, the promotion of the internationalization of the RMB and the diversification of foreign reserve investments are crucial steps in strengthening its financial stability. Additionally, establishing and effectively managing sovereign investment funds are crucial for mitigating financial risks. Following the downgrades by Moody’s and S&P, China must remain vigilant and implement appropriate policies to prevent the recurrence of severe financial disasters, including sovereign credit downgrades.

Despite providing empirical evidence for responding to a potential sovereign debt crisis in the post-COVID-19 era, this study has several limitations. The first limitation is the availability of data. This study collected from 73 countries from 2009 to 2016, with several indicators lacking data for specific years. In future research, more extensive data periods and an increase in the national data utilized would be beneficial for testing the validity of the results, especially regarding intra-sample grouping and inter-group heterogeneity. Secondly, the design of the study has room for improvement. For instance, principal component analysis (PCA) could be implemented instead of the manual empirical selection. Furthermore, using a dynamic panel data model, such as SYS-GMM, would be more effective in addressing potential issues such as sequential correlation, heteroscedasticity, and endogeneity, as opposed to using a static model. Thirdly, the Chinese and global economies are undergoing unprecedented transformations. Future research should consider incorporating elements of this dynamic transformation to gain a deeper understanding of the complex relationships between sovereign credit risk and economic, political, and social concerns.
